# Guidelines on the Role of Nursing in Patients with Chronic Inflammatory Arthritis: Do we “AGREE II”?

**DOI:** 10.31138/mjr.31.3.306

**Published:** 2020-09-30

**Authors:** Paraskevi Tsiamalou, Alexandros G. Brotis

**Affiliations:** 1 Department of Rheumatology, General University Hospital of Larissa, Larissa, Greece; 2 Department of Neurosurgery, General University Hospital of Larissa, Larissa, Greece

**Keywords:** guidelines, recommendations, nurse, chronic inflammatory arthritis, rheumatology

## Abstract

In the era of evidence-based practice, the role of the nurse in Rheumatology has broadened and became more complex, as a part of a multidisciplinary team. The European League Against Rheumatism (EULAR) has published recommendations for the role of the nurse in the management of chronic inflammatory arthritis in 2012, with an updated version in 2018. The objective of this study was to assess the methodological quality and reporting clarity of these recommendations using the AGREE II tool.

## INTRODUCTION

Chronic inflammatory arthritis (CIA) is a manifestation of a spectrum of systemic autoimmune disorders, such as rheumatoid arthritis, ankylosing spondylitis, and psoriatic arthritis that can severely impair physical function and quality of life.^1^ Familiarity with the presenting symptoms, including joint pain, swelling, and stiffness, is crucial in the early diagnosis and timely treatment.^1^ Patients with CIA are at greater risk for serious infections, respiratory disease, osteoporosis, cardiovascular disease, tumours, and death than the general population, due to the underlying disease or its treatment.^1^ This is true, despite the early diagnosis, aggressive treatment, and the use of disease-modifying anti-rheumatic drugs that have markedly improved both the management and long-term prognosis.^1^

Traditionally, the diagnosis and management of CIA are physician-oriented.^2,3^ The guidelines focus on the disease stratification and treatment allocation, mostly. The treatment options usually include non-steroidal anti-inflammatory drugs, glucocorticoids, oral small molecules, and biologic agents.^2,3^ However, these treatments are associated with important adverse events and increased costs.^4,5^ Recently, there was an increased interest in non-pharmacological modalities, such as physical and occupational therapy, lifestyle modification, and exercise, which were reserved for patients with minor symptomatology.^2,3^ The role of the nurse in the management of rheumatic disorders expanded to include self-management support, patient education and counselling, participation in multidisciplinary teams, prescription of drug treatments, and leading nurse-based clinics.^6,7^

The European League Against Rheumatism (EULAR) developed the recommendations for the role of the nurse in the management of patients with chronic inflammatory arthritis in 2012.^8^ In a first step, a Task Force made up of a multidisciplinary expert panel across Europe, identified the aims, formulated the research questions, chose the target population, and performed an extensive systematic literature review.^8^

Subsequently, the Task Force formulated the preliminary recommendations, and reached a consensus on 10 recommendations, a research agenda, and an educational agenda after discussion.^8^ In 2018, the EULAR recommendations were updated to include three overarching principles and eight recommendations.^9^

The primary objective of this study was to assess the reporting clarity and methodological quality of the EULAR recommendations for the role of the nurse in the management of CIA using the Appraisal of Guidelines for REsearch & Evaluation (AGREE) II tool.^8–10^ Secondary objectives were to document how these parameters evolved from the initial to the updated version, and identify potential areas for future improvement.^8,9^

## METHODS

The full-texts of the initial EULAR recommendations and the updated version, including their supplements were obtained and studied, thoroughly.^8,9^ Two reviewers, a registered nurse with more than 17 years of experience (TP) and a methodologist experienced with Bioinformatics and Biostatistics (AGB), co-operated for the review process. Both reviewers completed the AGREE II online training.^11^ None of them had participated in the writing or the development of EULAR recommendations.

The AGREE II tool consists of 23 items organized in five domains (scope and purpose, stakeholder involvement, rigor of development, clarity of presentation, applicability, and editorial independence) and two additional items (overall assessment). Each item was rated on a 7-point Likert scale (1, strongly disagree; 7, strongly agree). Final domain scores were calculated according to the AGREE II tutorial and sample test practice guideline.^11^ The two reviewers rated each domain, independently. The results were visualized in barplots side-by-side.

The scores of the 2012 recommendations and its 2018 update were compared using chi-square test for proportions. Meanwhile, the domain scores were categorized as high (≥80%), medium (60–79%), low (40–59%), or very low (≤40%). The degree of agreement between reviewers was determined by the measurement of intra-class correlation coefficient (ICC) and visualized by the correlation plot of the two reviewers. An ICC of > 0.9 was considered “very good”, between 0.71 and 0.9 “good”, between 0.51 and 0.7 “moderate”, between 0.31 and 0.5 “fair”, and <0.31 “poor” or “non-existent”. All statistical analyses were performed using Excel and the Real Statistics package.^12^ Statistical significance was considered for *p* values of less than 0.05.

## RESULTS

**Figure 1.**
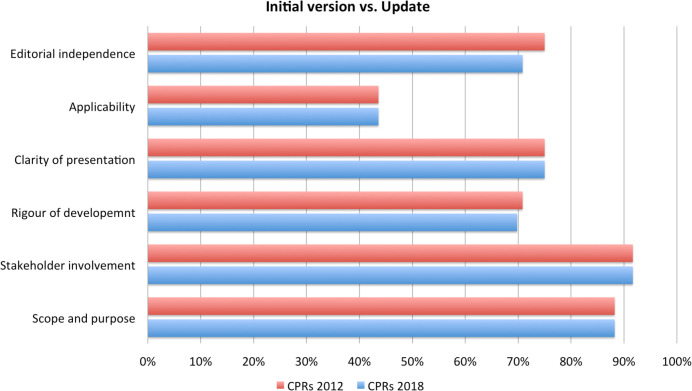
The bars compare the scores of the 2012 and 2018 EULAR recommendations on the role of the nurse in the management of the CIA using the AGREE II tool. (EULAR, European League Against Rheumatism; AGREE, Appraisal of Guidelines for research and Evaluation)

**Figure 2.**
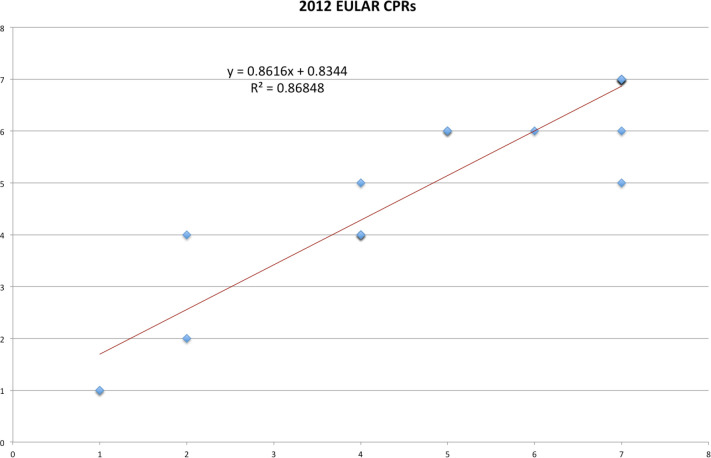
The AGREE II scores of the two reviewers for the 2012 EULAR recommendations on the role of the nurse in the management of the CIA are presented in a scatterplot. The correlation line and parameters of the correlation are depicted. (EULAR, European League Against Rheumatism; AGREE, Appraisal of Guidelines for research and Evaluation; CPRs, recommendations).

**Figure 3.**
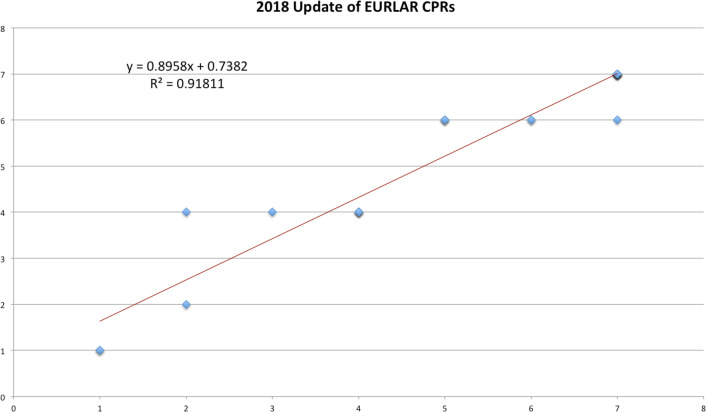
The AGREE II scores of the two reviewers for the 2018 EULAR updated recommendations on the role of the nurse in the management of the CIA are presented in a scatterplot. The correlation line and parameters of the correlation are depicted. (EULAR, European League Against Rheumatism; AGREE, Appraisal of Guidelines for research and Evaluation; CPRs, recommendations).

The AGREE II scores for the scope and purpose, stake-holder involvement, rigor of development, clarity of presentation, applicability, and editorial independence were 88%, 92%, 71%, 75%, 44%, and 75%, respectively (*Figure 1*, *Table 1*). The equivalent scores for the 2018 update (and the estimated differences between the two versions) were 88% (difference 0%; 95% CI ([−38.39%]- [38.39%]; *p*=1.0), 92% (difference 0%; 95% CI ([−37.52%]- [37.52%]; *p*=1.0), 70% (difference 1%; 95% CI ([−40.26%]- [40.26%]; *p*=0.968), 75% (difference 0%; 95% CI ([−40.53%]- [40.53%]; *p*=1.0), 44% (difference 0%; 95% CI ([−42%]- [42%]; *p*=1.0), and 71% (difference 4%; 95% CI ([−37.56%]- [43.95%]; *p*=0.871), respectively. In summary, the reporting clarity and methodological quality were high for domains 1 (scope and purpose) and 2 (stakeholder development), medium for domains 3 (rigor of development), 4 (clarity of presentation) and 6 (editorial independence), and poor for domain 5 (applicability). At the same time, the ICC for the 2012 recommendations and the 2018 update ranged from 50% to 93% and 88% to 99%, respectively (*Figure 2* and *Figure 3*).

**Table 1.** Table summarizing the appraisal of the EULAR recommendations of the role of nurses using the AGREE II tool.

		**Scope and Purpose**	**Stakeholder Involvement**	**Rigor of Development**	**Clarity of Presentation**	**Applicability**	**Editorial Independence**	**Overall**
**EULAR 2012**	Score	78	92	71	75	44	75	**Recommend**
	ICC	88	50	92	93	97	93	
**EULAR 2018**	Score	78	92	70	75	44	71	**Recommend**
	ICC	88	80	99	93	97	92	

(EULAR, European League Against Rheumatism; AGREE, Appraisal of Guidelines for research and Evaluation; ICC, intraclass correlation coefficient)

## DISCUSSION

This was the first study focusing on the methodological quality and reporting clarity of the EULAR recommendations on the role of the nurse in the management of CIA. Our findings were encouraging, as the scores were “high” and “medium” in most assessed parameters. On the contrary, the scores of the recommendations’ “applicability” were not as high. These results remained unchanged after the 2018 update.

Our results were in accordance to the evidence from the relevant literature. In a similar study, Duarte-García et al. used the AGREE II instrument to appraise the guidelines in the realm of Rheumatology from the physician’s perspective.^13^ The authors focused on nine relevant guidelines.^13^ The scores ranged between 78–99 for “scope and purpose”, 57–99 for “stakeholder involvement”, 87–96 for “rigor of the methodology”, 83–99 for “clarity of presentation”, 49–78 for “applicability”, and 69–85 for “editorial independence”.^13^ Over time, although the average domain quality of the guidelines improved for all, the “applicability” and “editorial independence” domains had the least amount of improvement.^13^

Van Eijk-Hustings conducted a multinational Web-based survey among 967 nurses, 548 rheumatologists, and 2034 patients from 23 countries to assess the level of agreement and application of the EULAR recommendations in 2014.^14^ Both agreement and application were assessed using a 0–10 Likert scale (0 = none, 10= full agreement/application).^14^ The median level of agreement was high in all three groups, whereas, the median level of application was substantially lower.^14^ Agreement and application were lowest in Eastern and Central Europe.^14^ The most commonly reported reasons for incomplete agreement were the too many “other responsibilities” according to the nurses, doubts about the knowledge of the nurse by the rheumatologists, and fear of losing contact with the rheumatologist according to the patients.^14^ The EULAR recommendations were characterized by significant strengths. The overall objectives of the recommendations have been presented, adequately. Meanwhile, the target audience has been fully described. All parts involved were represented, sufficiently. Professionals working in the field of Rheumatology (rheumatologists, nurses, and other disciplines), patients and policymakers were defined as the target users. The views of the target population have been sought and registered. The development of the EULAR recommendations followed a systematic literature search of evidence, with clearly described criteria for selecting the evidence. All different alternatives for management of the CIA by the nurses were clearly presented. Without any doubt, the key recommendations were easily identifiable in both versions. The 2012 recommendations have been updated to include all new knowledge up to the time of publication. Finally, the competing interests of the guideline development group have been recorded.

At the same time, there were a few issues in the current recommendations that have to be addressed in future versions. The health questions covered by the recommendations were not clearly defined. The 2012 version stated that the systematic literature search was based on eight questions, without any further reference. The strengths and limitations of the body of evidence and the methods for formulating the recommendations were clearly described, but the risk of bias due to the diversity of the eligible studies cannot be overemphasized. The health benefits, side effects, and risks have not been fully considered in formulating the recommendations. A link between the recommendations and the supporting evidence can be traced in the discussion section, but a table with explicit links between the recommendations and the relevant literature would be more efficient. Neither the 2012 recommendations nor its 2018 update was externally revised. With regards to the clarity of presentation, the recommendations have been unambiguous; nevertheless, they lack specificity. Of note, the domain “applicability” recorded the lowest scores. Only a few of the barriers and facilitators have been addressed. The recommendations did not provide advice on how the recommendations can be put into practice. Furthermore, the potential resource implications have not been fully considered, and there were no monitoring and auditing criteria. Regarding the “editorial independence”, it was not clear if the EULAR, which is the funding body, has influenced the content of the recommendations.

In conclusion, the EULAR recommendations, in addition to their significance in the clinical setting, were soundly developed and clearly presented. Future versions need to address a few important issues, with particular interest on the validation of the recommendations by an external reviewer, applicability of the recommendations, and editorial independence.

## ABBREVIATIONS

AGREE: Appraisal of Guidelines for REsearch & Evaluation
CIA: Chronic inflammatory arthritis
EULAR: European League Against Rheumatism
ICC: Intraclass correlation coefficient
